# MicroRNA-107-5p suppresses non-small cell lung cancer by directly targeting oncogene epidermal growth factor receptor

**DOI:** 10.18632/oncotarget.18505

**Published:** 2017-06-16

**Authors:** Ping Wang, Xiaomin Liu, Yang Shao, Huimin Wang, Chen Liang, Baohui Han, Zhongliang Ma

**Affiliations:** ^1^ Laboratory for Noncoding RNA & Cancer, School of Life Sciences, Shanghai University, Shanghai, China; ^2^ Cancer Institute, Fudan University Shanghai Cancer Center, Department of Oncology, Shanghai Medical College, Fudan University, Shanghai, China; ^3^ Department of Pulmonary Medicine, Shanghai Chest Hospital, Shanghai Jiao Tong University, Shanghai, China

**Keywords:** MicroRNA-107-5p, EGFR, NSCLC, tumorigenesis, proliferation

## Abstract

MicroRNAs (miRNAs) are dysregulated in cancers, including human non-small cell lung cancer (NSCLC). The function of MicroRNA-107-5p (miR-107-5p) in NSCLC is not fully elucidated. Epidermal growth factor receptor (EGFR) is a cancer-driven gene in tumorigenesis. In this study, we found that miR-107-5p was significantly decreased in NSCLC tissues and NSCLC cell lines. Moreover, our results indicated that miR-107-5p could suppress cell proliferation, inhibit metastasis, impede cell cycle, and promote apoptosis via directly targeting EGFR. We also investigated roles of miR-107-5p *in vivo*. The results showed that it could inhibit tumor growth. Therefore, our study demonstrated that miR-107-5p not only suppressed the progression in NSCLC cells by inhibiting the expression of EGFR, but also could be a promising and a new potential therapeutic target for lung cancer.

## INTRODUCTION

Lung cancer is one of the most prevalent malignancies and the most common cause of cancer-related death in the worldwide [1]. About 80% of lung cancer patients suffered from non-small cell lung cancer (NSCLC). Fortunately, the incidence rate of NSCLC is decreasing with improvement of early diagnosis and clinical treatment strategies but the 5-year survival rate is still very poor [2, 3]. More and more studies have shown that microRNAs (miRNAs) are involved in lung carcinogenesis, providing an important treatment option for patients [4, 5].

EGFR is a member of the human epidermal growth factor receptor (HER) family, acting as a proto-oncogene which is releated to cell proliferation, metastasis, and tumorigenesis [6–8]. EGFR abnormal expression occurs in many cancer patients mostly due to its mutation, especially in NSCLC [9]. Tyrosine kinase inhibitors specific to EGFR (EGFR TKIs) are used in patients with NSCLC such as Gefitinib, Erlotinib and Osimertinib, however the limitation of this treatment is the EGFR gene-mutation status, drug resistance to EGFR-TKIs presents a major issue [10].

MiRNAs are a class of 18-24 nt non-coding RNA molecules through to primarily regulate cellular function by binding to the 3′-untranslated region (3′-UTR) of mRNA [11–13]. MiRNAs function as key factors in predicting disease status and prognosis in cancers [14, 15]. On the one hand, miR-107 played vital function in stress response, metabolism, and cell division [16]. On the other hand, miR-107 was associated with several pathological conditions, such as neurodegenerative disease, angiogenesis and tumorigenesis. Dysregulated of miR-107 is related to various tumor development [17, 18].

In view of the fact that miR-107-5p is significantly down regulated in NSCLC, moreover, we hypothesized that miR-107-5p may play an important role in tumorigenesis and tumor development in NSCLC by regulating EGFR.

## RESULTS

### MiR-107-5p is downregulated in NSCLC

**Figure 1:**
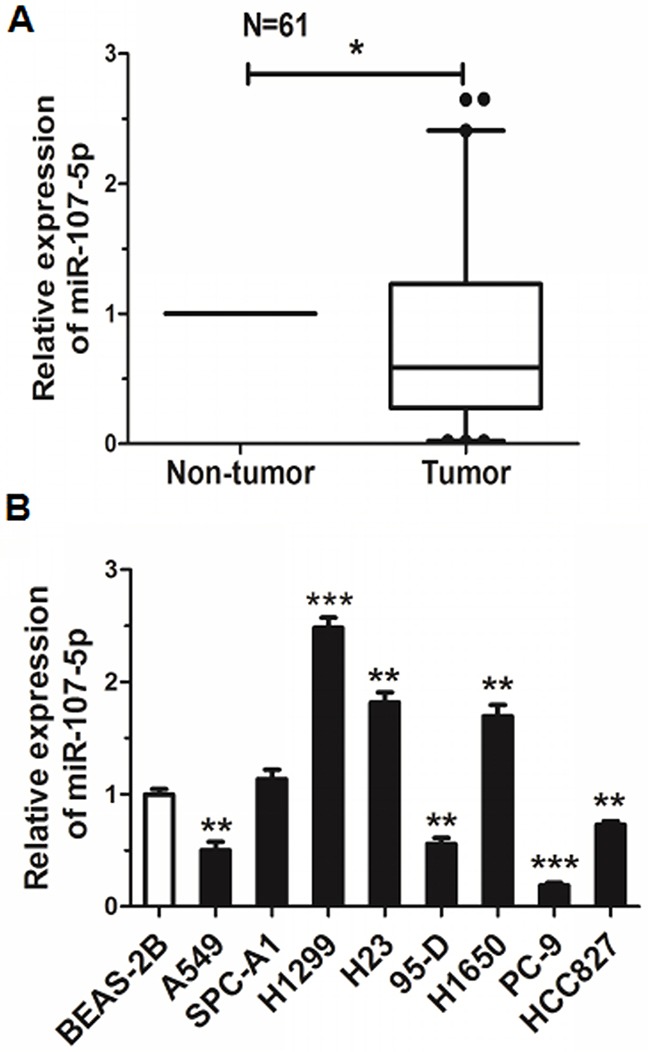
Expression of miR-107-5p decreases in NSCLC **(A)** Expression of miR-107-5p in NSCLC and corresponding non-tumor tissues (*n* = 61). **(B)** Expression of miR-107-5p in the human NSCLC cell lines and BEAS-2B (normal control) was measured by qRT-PCR (*P < 0.05, **P < 0.01, ***P < 0.001).

To investigate the role of miR-107-5p in lung cancer, we first analyzed the expression level of miR-107-5p in 61 human NSCLC samples and para-carcinoma tissues. Our data revealed that miR-107-5p was down regulated when compared with corresponding para-carcinoma tissues (Figure 1A). The expression of miR-107-5p in NSCLC cell lines was also examined, and miR-107-5p was significantly down regulated in A549, 95-D, PC-9, HCC827 cells when compared with BEAS-2B cells, the normal bronchial epithelial cells (Figure 1B). These results suggested that the miR-107-5p was associated with NSCLC carcinogenesis.

### MiR-107-5p inhibits cell proliferation and migration

**Figure 2:**
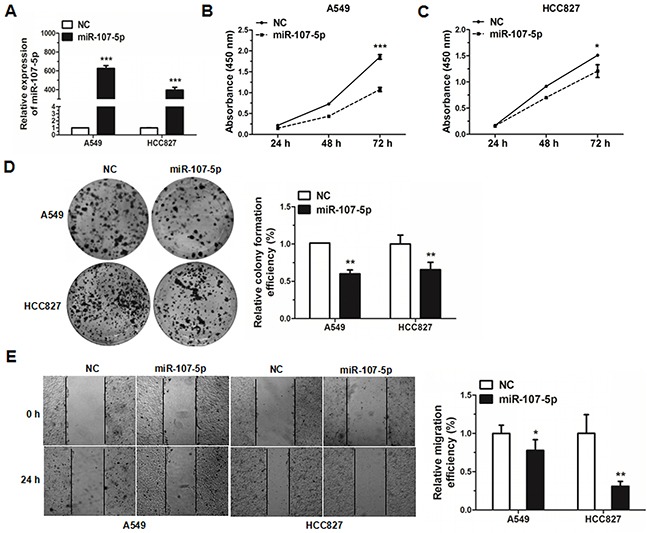
MiR-107-5p inhibits cell proliferation and migration in NSCLC cells **(A)** The expression of miR-107-5p was measured by qRT-PCR in A549 and HCC827 cells 48 h after transfection with NC (negative control) or miR-107-5p mimic. **(B-C)** A549 and HCC827 cells were transfected with NC or miR-107-5p mimic, and cell proliferation was determined by CCK-8. **(D)** Colony formation assay was performed in A549 and HCC827 cells after transfected with NC or miR-107-5p mimic. **(E)** Wound-healing assays were performed to evaluate the effect of miR-107-5p on the migratory ability in the A549 and HCC827 lines. Photos of the cells were taken at 0 and 24 h, the relative migration length was from three randomly selected locations. All experiments were repeated in triplicate (*P < 0.05, **P < 0.01, ***P < 0.001).

A549 cells, HCC827 cells and 95-D cells were transfected with miR-107-5p mimic. The transfection of miR-107-5p increased its level in these cell lines by qRT-PCR analysis (Figure 2A, Supplementary Figure 1A). The effect of miR-107-5p on proliferation was assessed by Cell Counting Kit-8 (CCK-8) assays and cell colony formation assays (Figure 2B–2D, Supplementary Figure 1B–1C). The CCK-8 and cell colony formation assay showed that there was a significant decrease in the absorbance after transfection with miR-107-5p mimic in A549 cells, HCC827 cells and 95-D cells when compared with the negative control (NC) group.

In order to better understand miR-107-5p function in human NSCLC cell metastasis, wound-healing assays were experimented in A549 and HCC827 cell lines. The results revealed that expression of miR-107-5p decreased the migration rate of A549 and HCC827 cell lines when compared with the NC group (Figure 2E). Together, the data demonstrated that miR-107-5p can inhibit cell proliferation and migration of NSCLC cells.

### MiR-107-5p promotes apoptosis and impedes cell cycle

**Figure 3:**
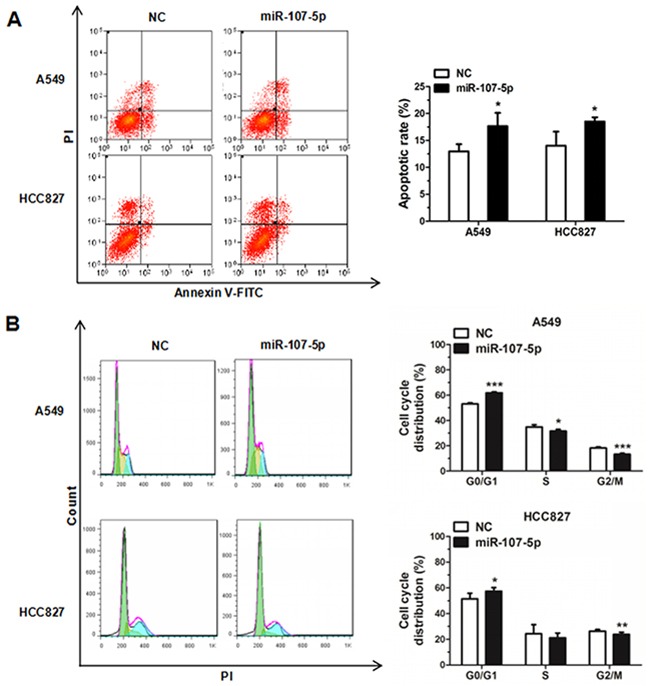
MiR-107-5p promotes apoptosis and inhibits cell cycle progression in NSCLC cells Cell cycle analysis was analyzed on A549 and HCC827 cells by flow cytometry. **(A)** Cell apoptosis situations of A549 and HCC827 cells transfected with NC or miR-107-5p mimic were increased by flow cytometry. **(B)** The cell cycle distributions of A549 and HCC827 cells transfected with NC or miR-107-5p mimic were detected by flow cytometry. All experiments were repeated in triplicate (*P < 0.05, ** P<0.01, *** P<0.001).

A549 cells, HCC827 cells and 95-D cells were treated with miR-107-5p mimic for 48 h and then were stained with FITC/PI and analyzed by flow cytometry. The percentages of apoptotic cells in the miR-107-5p mimic groups were significantly higher than that of NC group (Figure 3A, Supplementary Figure 1D). To determine the role of miR-107-5p on cell cycle, cells were transfected with miR-107-5p mimic for 24 h, and the results analyzed by flow cytometry. The results revealed that the NSCLC cells were arrested in G0/G1 phase (Figure 3B, Supplementary Figure 1E). These data suggested that miR-107-5p could promote the level of apoptosis in NSCLC cells and arrest cells in the G1 phase and reduced the proportion of cells in the S phase. Thus miR-107-5p promotes apoptosis and arrests cell cycle in NSCLC cells.

### EGFR is a direct target of miR-107-5p

**Figure 4:**
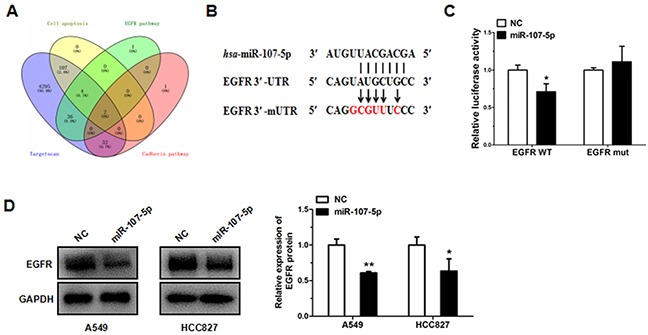
EGFR is a direct target of miR-107-5p **(A)** Identification of the candidate target of miR-107-5p by bioinformatics. Blue: the predicted targets generated by targetscan. Yellow: the genes involved in apoptosis. Green: the genes involved in EGFR signaling pathway. Pink: the genes involved in cadherin signaling pathway. **(B)** Schematic of the seed region of miR-107-5p in the 3′-UTR of EGFR (EGFR-3′-UTR) and the mutated 3′-UTRs (EGFR-3′-mUTR) as predicted by bioinformatics. **(C)** There was a significant decrease in the luciferase activity of HEK293T cells co-transfected with miR-107-5p mimic and pGL3-EGFR-3′-UTR. **(D)** Gene expressions were measured by western blot in A549 and HCC827 cells transfected with NC and miR-107-5p mimic. All experiments were repeated in triplicate (*P < 0.05, ** P<0.01, *** P<0.001).

To identify the potential target genes of miR-107-5p, bioinformatics method (www.targetscan.org) was applied in this study. MiR-107-5p has been predicted to be related to apoptosis, metastasis, and EGFR pathways [19, 20]. We found that the EGFR was in the intersection through using Venn diagram (Figure 4A). EGFR was selected as a potential target. The wild type binding site of miR-107-5p with EGFR was displayed by using in TargetScan prediction programs and the mutant type binding site of miR-107-5p with EGFR were also shown (Figure 4B).

In order to validate whether EGFR is a direct target of miR-107-5p, we constructed two recombinant expression vectors containing the miR-107-5p wild type binding sequences in the 3′UTR of EGFR and mutated five binding sites within the 3′UTR (pGL3-EGFR-3′-UTR, and pGL3-EGFR-3′-mUTR), and co-transfection of miR-107-5p mimic or NC mimic in HEK293T cells with pGL3-EGFR-3′-UTR resulted in significant restraint of luciferase activity (co-transfected with pGL3-EGFR-3′-UTR and miRNA NC). Moreover, it had no difference when compared with negative control while co-transfected with pGL3-EGFR-3′-mUTR and miR-107-5p mimic in the HEK293T cells (co-transfected with pGL3-EGFR-3′-mUTR and miRNA NC) (Figure 4C). The protein expression of EGFR was reduced in A549 cells and HCC827 cells after transfected with miR-107-5p mimic (Figure 4D).

### Downregulation of EGFR reduces the progress of NSCLC

**Figure 5:**
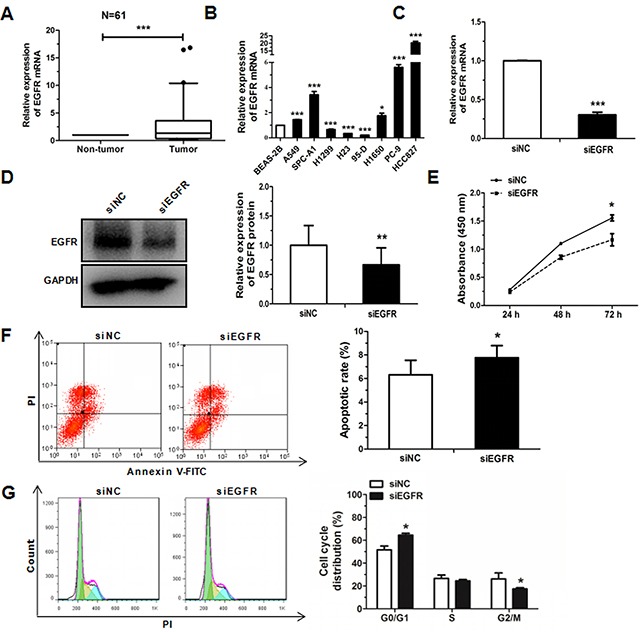
Knockdown of EGFR inhibits cell proliferation, cell cycle and promote apoptosis in HCC827 cell lines **(A)** Relative expression of EGFR in NSCLC and corresponding non-tumor tissues (*n* = 61). (**B**) Expression of EGFR in the human NSCLC cell lines and a BEAS-2B was measured by qRT-PCR. **(C)** EGFR mRNA was detected by qRT-PCR in HCC827 cells transfected with EGFR siRNA or siNC. **(D)** The protein levels of EGFR were detected by western blot in HCC827 cells transfected with EGFR siRNA or siNC. **(E)** The proliferation of HCC827 cells transfected with EGFR siRNA or siNC, as determined by CCK-8 assay. **(F)** Cell cycle distribution was analyzed by flow cytometry in HCC827 cells transfected with EGFR siRNA or siNC. **(G)** Cell apoptosis distribution was analyzed by flow cytometry in HCC827 cells transfected with EGFR siRNA or siNC. Each experiment was performed in triplicate at least (*P < 0.05, **P < 0.01, ***P < 0.001).

In order to determine the role of EGFR, the same 61 pairs of NSCLC patient tissues were detected by qRT-PCR. Our results revealed that the expression of EGFR was significantly decreased, as compared with their corresponding para-carcinoma tissues (Figure 5A). Meanwhile, we detected EGFR RNA levels in the same NSCLC cell lines by qRT-PCR. Our results revealed that the expression of the EGFR was much higher than BEAS-2B control cells in NSCLC cell lines (Figure 5B).

To further explore the biological effects involved in regulation of EGFR expression by miR-107-5p in NSCLC cell lines, EGFR was knocked down by using siRNA to test the change in cell proliferation, cell cycle and apoptosis progression of A549 cells and HCC827 cells. Cells were transfected with siEGFR after 48 h, we found the expression of EGFR mRNA was significantly decreased as compared to the control and the expression of protein has the same trend (Figure 5C–5D). Moreover, cell proliferation, cell cycle, and cell apoptosis were determined in HCC827 cells following treatment with siEGFR. The results of CCK8 showed that the proliferation ability of NSCLC cells decreased significantly compared with NC group (Figure 5E). Cell cycle analysis revealed that the proportion of cells at G0/G1 phase increased as compared with the control when treated with siEGFR for 48 h (Figure 5F). In addition, it also could promote apoptosis compared with the control (Figure 5G).

### MiR-107-5p suppresses the tumorigenesis *in vivo*

**Figure 6:**
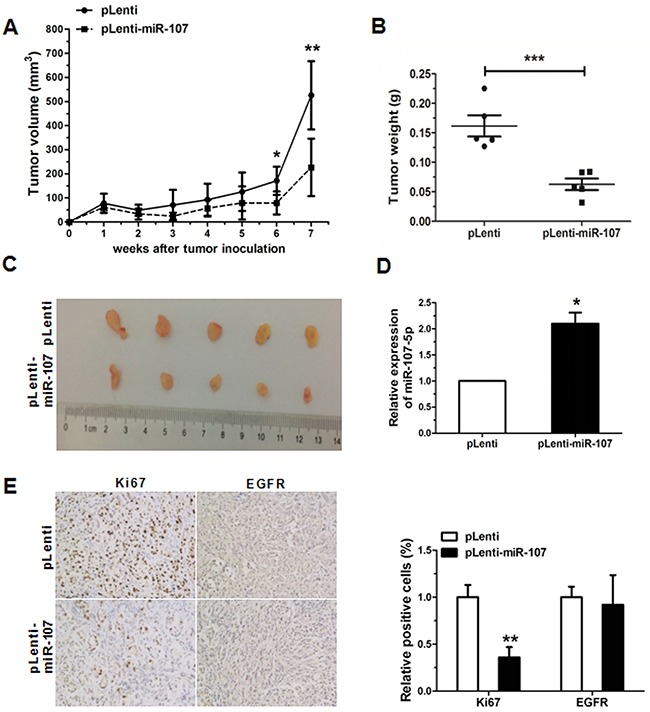
Overexpression of miR-107 inhibits tumor growth **(A)** MiR-107-stably-overexpressing A549 cells (pLenti-miR-107) and control (pLenti) were injected into nude mice. Each group comprised 5 mice. Tumor sizes were measured every week and growth curves were generated. **(B)** Mice were sacrificed after 7 weeks and tumor weights were measured. **(C)** Tumor images were displayed. **(D)** The expression of miR-107 was detected by qRT-PCR in the mouse tumor tissues induced by NC and miR-107-stably-overexpressing A549 cells. **(E)** The expression of Ki67, EGFR in tumor tissues was measured by immunohistochemistry (*P < 0.05, **P < 0.01, ***P < 0.001).

To explore the role of miR-107-5p in tumor proliferation *in vivo*, nude mice were treated with A549 cells (5 × 10^6^) with stably overexpressing miR-107 (pLenti-miR-107) or control (pLenti). The tumor size was measured every week, and the growth curve against the average tumor size was plotted. After 7 weeks, all of the mice were sacrificed according to the protocol, and the xenografts were weighed. There was a significant decrease in tumor size and weight of the miR-107-overexpressing groups compared with the control (Figure 6A–6C). Meanwhile, the expression of miR-107-5p was significantly upregulated in tumor tissues of pLenti-miR-107 group compared to the control (Figure 6D). Immunohistochemical analysis of tumor tissues from miR-107-stably-overexpressing mice, and results showed that Ki67 was significantly downregulated (Figure 6E). Taken together, these presentations suggested that miR-107-5p is a negaitive regulator in NSCLC.

## DISCUSSION

Emerging evidence has demonstrated that miRNAs play key roles in tumorigenesis. Some miRNAs can target oncogenes, acting as tumor suppressors in tumors, while others act as tumor promoters by targeting anti-oncogenes [21–23]. MiR-107-5p has been demonstrated to act as an oncogene in hepatocellular carcinoma and gastric cancer [19, 24]. In contrast, miR-107-5p functions as a tumor suppressor in colorectal cancer, glioma and NSCLC [25–27]. It is urgent to understand miR-107 in lung cancer.

In this study, we demonstrated that in clinical NSCLC samples, miR-107-5p was significantly downregulated compared with the control, suggesting its role as a tumor suppressor. A549 cells and HCC827 cells belong to adenocarcinoma, especially, that HCC827 had deletional mutations (E746-A750) in EGFR exon 19 which is different from other cell lines that could help us to discuss the importance of EGFR. Our results showed that miR-107-5p can inhibit cell proliferation, colony formation, metastasis, cause cell cycle arrest at G0/G1 phase and promote apoptosis in A549, HCC827 and 95-D cells. Additionally, the *in vivo* experiments were also performed, the results revealed that tumor growth was significantly inhibited, compared to the control.

**Figure 7:**
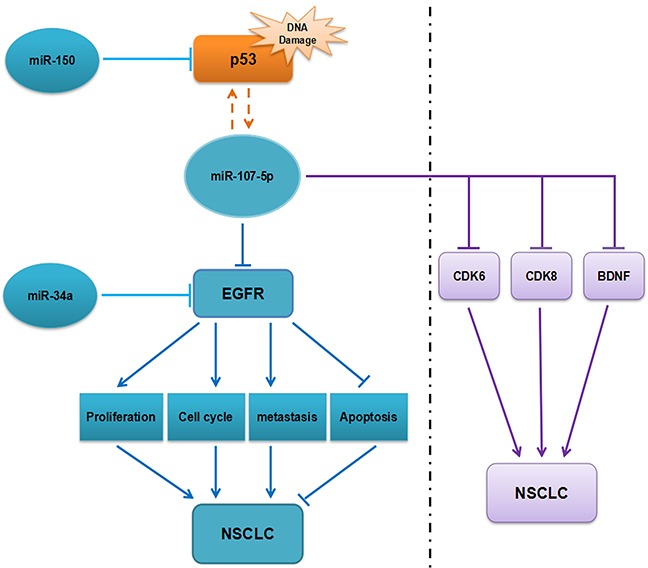
The regulatory network of miR-107-5p in NSCLC We speculate that miR-107-5p function as tumor suppressor by down-regulating EGFR and has a close correlation with several miRNAs or genes that are related to the NSCLC process.

Our results demonstrated that EGFR was a direct target of miR-107-5p in NSCLC cell lines for the first time, and based on previous studies in our lab, we found that miR-150 [28], miR-34a and miR-107-5p may have a close correlation with NSCLC development by partly targeting EGFR. Meanwhile, miR-107-5p has also been proved to target other genes, such as over-expression of miR-107 could arrest cell cycle and suppress cell proliferation in H1299 and A549 cells by targeting CDK6, miR-107 could directly target CDK8 to further inhibit cell proliferation in A549 cells and miR-107 could inhibit tumor growth and metastasis in NSCLC cells by directly targeting BDNF and regulating the P13K/AKT signaling pathway indirectly [26, 29, 30] (Figure 7). There should be a complex regulatory network in cell activities. In brief, EGFR can be regulated by multiple miRNAs and genes, the mechanism is worth to study thoroughly. Our results validate that miR-107-5p functions as a tumor suppressor through EGFR, and this shed light on precision medicine of lung cancer.

## MATERIALS AND METHODS

### Tissue samples

All lung cancer tissue samples were obtained from the Department of Oncology, Shanghai Chest Hospital (Shanghai, China), and approved by the Ethics Committee of Shanghai Hospital and patients. The details of the samples used in this paper are listed in Supplementary Table 1.

### Cell culture and cell transfection

SPCA-1, 95-D, HEK293T, and BEAS-2B cells were obtained from the Cell Bank, China Academy of Sciences (Shanghai, China). A549, H1650, HCC827, H23 and H1299 cells were purchased from the American Type Culture Collection (ATCC, Manassas, VA, USA). The NSCLC cell lines, HCC827, H1299, H1650 and H23 were cultured in RPMI-1640 medium. A549, HEK293T, BEAS-2B, 95-D and SPCA-1 cells were cultivated in DMEM (Gibco). Cells were cultured in 90% media, 10% fetal bovine serum (FBS, HyClone Laboratories, Logan, UT, USA), 100 μg/ml penicillin, 100 μg/ml streptomycin (Gibco), and antibiotic cocktail. HCC827 cells were cultured with 20% fetal bovine serum. All cells were cultured at 37°C in a 5% CO_2_ humidified environment. BEAS-2B is the normal bronchial epithelial cells. 95-D is belonging to large cell carcinoma, high metastatic lung cancer. HCC827, H1650 and PC-9 are belonging to adenocarcinoma, EGFR gene-mutation cell lines. A549, H1299, H23 and SPC-A1 are belonging to adenocarcinoma, EGFR gene-wild cell lines.

Cells were transiently transfected with 30 nM of miR-107-5p mimic, negative control mimic (NC), and transfected with 150 nM EGFR siRNA (siEGFR), or negative control siRNA (siNC) (RIBOBIO, Guangzhou, China) using Invitrogen™ Lipofectamine 2000 (Life Technologies, New York, USA) according to the manufacturer's instructions. After 24 h to 48 h post-transfection, cells were used for qRT-PCR, cell proliferation, colony formation, cell cycle analysis, apoptosis analysis and western blot.

### qRT-PCR analysis

Total RNA extracted from cells by using TRIzol Reagent (Sangon Biotech, Shanghai, China). Reverse transcription was performed using the PrimeScript^TM^ 1st Strand cDNA Synthesis Kit (M-MLV RTase cDNA Synthesis Kit, TaKaRa, Dalian, China). Meanwhile, the PrimeScript®miRNA First-Strand cDNA Synthesis SuperMixQuantiMir cDNA Kit (Transgen Biotec, Beijing, China) was used to synthesize a cDNA library of miRNAs. The level of mRNA or miRNA was quantified by qRT-PCR using a SYBR Green PCR master mix (TaKaRa). The endogenous controls for mRNA and miRNA were 18S RNA and U6 snRNA, respectively. Results were expressed using relative quantification (^2-ΔΔCt^) method. All primer sequences were listed in Supplementary Table 2.

### Cell proliferation assay

Cells were plated at 96-well plate with a density of 2×10^3^ or 4×10^3^cells per well, CCK-8 was added and returned to incubation conditions for 2–4 h. Light absorbance at 450 nm was measured daily with a microplate reader. All experiments were triplicates.

### Colony formation assay

Cells were plated at 300 or 600 cells per well in 6-well plates and incubated for 2 weeks at 37°C in a 5% CO_2_ humidified environment. Colonies were stained with crystal violet (0.5% w/v) after fixed with methanol, and then counted. Experiments with duplicates were performed.

### Cell cycle and apoptosis analysis

Cell cycle analysis and apoptosis were performed as previously described [31]. Cells (10^6^/ml) were seeded in 6-well plates and transfected after 24-48 h to culture, and then cells were subjected to an annexin V/ propidium iodide (PI) or propidium iodide (PI) according to the manufacturer's protocol. Thencells were analyzed distribution with a MoFlo XDP flow cytometer (Beckman Coulter, Inc., Brea, CA, USA). The analysis of data was using Flow Jo software (Treestar Inc., USA). Experiments with duplicates were performed.

### Wound-healing assay

For cell metastasis assay, cells were seeded in 6-well plates transiently transfected with miR-107-5p mimic, and NC group. Then cells were incubated in their respective complete culture medium until grown to confluence. Single scratch wounds were created using a 200 μl tip, then using serum-free medium to remove the debris. Finally, serial photographs were taken at different time points.

### Dual luciferase reporter assay

Dual luciferase reporter assay was tested as previously showed [31]. In brief, the 3′-UTR of the target gene (EGFR) was amplified and inserted downstream of the firefly luciferase reporter gene in the pGL3 miReport vector (Promega, Madison, WI, USA), named pGL3-EGFR-3′-UTR listed in Supplementary Table 2. Meanwhile, the sequences of the mutated EGFR were Constructed which named pGL3-EGFR-3′-mUTR listed in Supplementary Table 2, and confirmed by sequencing (Sangon Biotech, China). HEK293 cells were cultured in 24-well plates to measure luciferase activity. When grown to 60–80% confluence, the cells were transiently co-transfected with 400 ng of luciferase vector pGL3-EGFR-3′-UTR or pGL3-EGFR-3′-mUTR, and miR-107-5p mimic or NC miRNA, we used 100 nM with 20 ng plasmid expressing the renilla luciferase gene (pRL, Promega) as a final concentration for transfection efficiency which was the control. After incubation for 48 h at 37°C, the luciferase activity was determined using an Orion II Microplate Illuminometer (Titertek-Berthold, South San Francisco, USA).

### Western blot analysis

Western blot analysis was performed as previously described [31]. To be short, cellular proteins were extracted from cultured cells using RIPA lysis buffer (CWBIO, Beijing, China) and using a Protein BCA Assay Kit (Bio-Rad, Hercules, California, USA) to quantify content of protein. Protein samples were separated by sodium dodecyl sulfatepolyacrylamide gel electrophoresis (SDS-PAGE) and transferred to a polyvinylidene difluoride (PVDF) membrane (Millipore Corporation, Billerica, MA, USA). After blocking in 5% powdered milk at room temperature for at least 1 h, the membranes were incubated with rabbit anti-EGFR, and anti-GAPDH antibodies (1:1000, Cell Signaling Technology, Danvers, MA, USA) overnight at 4°C. After washing and incubation, goat-anti-rabbit as the secondary antibodies were conjugated to horseradish peroxidase (HRP) (1:1000, Cell Signaling Technology). Subsequent visualization was detected with a chemiluminescent HRP substrate (Millipore Corporation, Billerica, USA) and imaged with an E-Gel Imager.

### Lentivirus construction and infection

Performed as previously described [32], pri-miR-107 was amplified in A549 cells, then digested with *Xho* I and *Bam*H I and cloned into the lentiviral expression vector, pLenti (Invitrogen, Carlsbad, CA, USA), named pLenti-miR-107. We confirmed the recombinant expression vector by sequencing (Sangon Biotech, China). Next, pLenti vector or pLenti-miR-107 was co-transfected into HEK293T cells using a packaging plasmid system (pMD2G and psPAX2) and viral particles, after 24 h and 48 h later to collect lentiviral particles. Then A549 cells were infected with the viral particles twice within 48 h. Transfected green fluorescence to pLenti vector or pLenti-miR-107 in A549 cells. Flow cytometry (Beckman Coulter) was used to obtain stable overexpression of miR-107 (pLenti-miR-107) or negative control (NC, pLenti) in A549 cells. Finally, cells were cultuerd for further experiments.

### Animal studies

Female nude mice (five-week-old) were purchased from the SLRC Laboratory Animal Center (Shanghai, China). After 1 week mice were randomly assigned into 2 groups, and 5 × 10^6^ A549 cells stably overexpressing miR-107 (pLentimiR-107) or control (pLenti) were injected into each mouse. Tumors were measured once a week, and the tumor volume was calculated using the following formula: volume = length × width^2^/2. Tumors were resected and weighed. All animal experiments were performed according to the animal experimental protocols approved by the institutional Animal Care and Use Committee of Shanghai University (Shanghai, China).

### Immunohistochemcal

It was performed as previously described [32]. Tumor biopsies were fixed by formalin, embedded by paraffin, and finally cut in sections of about 4 μm. Samples were rehydrated with demineralized water after deparaffinized and dehydrated with xylene and graded alcohols. We using microwave pre-treatment of slides for antigen retrieval to perform immunohistochemistry. The primary antibodies against Ki67, EGFR (1:500, Cell Signaling Technology) were used, and the proteins *in situ* were visualized by 3, 3′-diaminobenzidine reaction solution after together with goat anti-rabbit horseradish peroxidase (HRP)-conjugated antibodies.

### Statistical analysis

Results were expressed as group means ± SEM and analyzed with student′s *t*-test for 2-group comparisons. Differences were considered statistically significant when *p* < 0.05.

## SUPPLEMENTARY MATERIALS


