# Supplementary material for: MicroRNA-107-5p suppresses non-small cell lung cancer by directly targeting oncogene epidermal growth factor receptor
Source: Oncotarget. 2017 Jun 16;8(34):57012–23. doi: 10.18632/oncotarget.18505 (PMC5593621; doi:10.18632/oncotarget.18505)
Supplement: Supplementary file 1 [file oncotarget-08-57012-s001.pdf]

MicroRNA-107-5p suppresses non-small cell lung cancer by directly targeting oncogene epidermal growth factor receptor

SUPPLEMENTARY MATERIALS

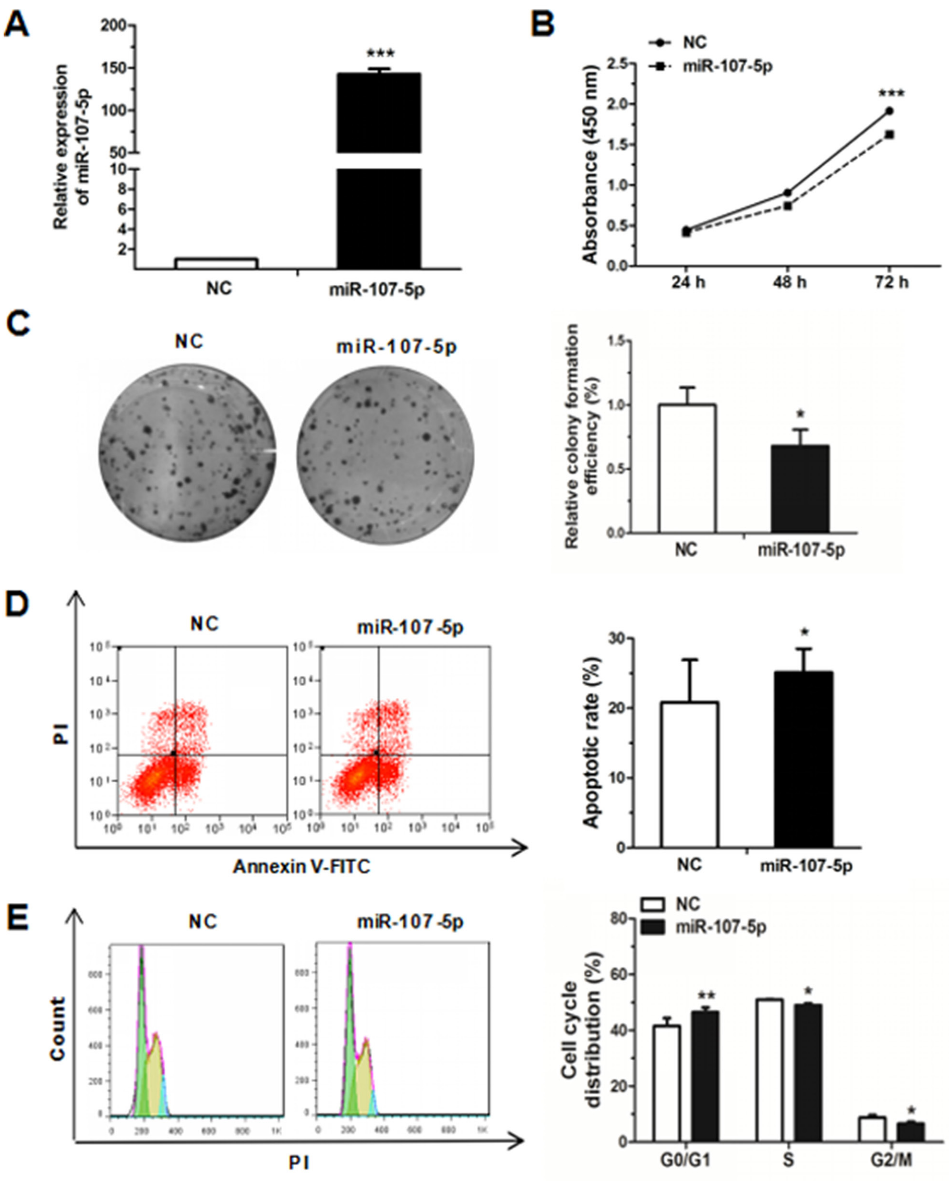

Supplementary Figure 1: MiR-107-5p suppresses cell proliferation, cell cycle and promotes apoptosis in 95-D cell lines. **(A)** The expression of miR-107-5p was measured by qRT-PCR in 95-D cells 48 h after transfection with NC (negative control) or miR-107-5p mimic. **(B)** 95-D cells were transfected with NC or miR-107-5p mimic, and cell proliferation was determined by CCK-8. **(C)** Colony formation assay was performed in 95-D cells transfected with NC or miR-107-5p mimic. **(D)** Cell apoptosis situations of 95-D cells transfected with NC or miR-107-5p mimic were increased by flow cytometry. **(E)** The cell cycle distributions of 95-D cells transfected with NC or miR-107-5p mimic were detected by flow cytometry. All experiments were repeated in triplicate (**P* < 0.05, ** P<0.01, *** P<0.001).

Supplementary Table 1: The clinical-pathological features of 61 cases NSCLC patients

See Supplementary File 1

Supplementary Table 2: PCR primers

Primer name	Primer sequences(5′-3′)
18S RNA(F)	AGGAATTCCCAGTAAGTGCG
18S RNA(R)	GCCTCACTAAACCATCCAA
U6 snRNA(F)	CTCGCTTCGGCAGCACA
U6 snRNA(R)	AACGCTTCACGAATTTGCGT
EGFR-3′-UTR(F)	GCTCTAGAGCGAGCGTTAGACTGACTTGTTTG
EGFR-3′-UTR(R)	GGAATTCCAGCGGTGCTATCCTTAGGTATT
EGFR-3′-mUTR(F)	AGTAAGGTCCCCCTGTCTTGCTGTCATGAA
EGFR-3′-mUTR(R)	CAGGGGGACCTTACTGAGTTTCAAAACAATG
EGFR(F)	CGCATTAGCTCTTAGACCCA
EGFR(R)	AGTAAGTACCGTGGGGACAT
pri-miR-107(F)	CGGGATCCCGAATGTGAGGAAAAGGTCTGAGC
pri-miR-107(R)	CCGCTCGAGCGGCCTCAACTCCTCTTTCCTGTCT
miR-107-5p	AGCAGCATTGTACAGGGCTATCA
